# Supplementary material for: The trait-specific timing of accelerated genomic change in the human lineage
Source: Cell Genom. 2025 Jan 8;5(1):100740. doi: 10.1016/j.xgen.2024.100740 (PMC11770217; doi:10.1016/j.xgen.2024.100740)
Supplement: Document S1. Figures S1–S11 [file mmc1.pdf]

Cell Genomics, Volume *5*

Supplemental information

The trait-specific timing of accelerated genomic change in the human lineage

Eucharist Kun, Mashaal Sohail, and Vagheesh M. Narasimhan

Supplemental Information
The trait specific timing of accelerated genomic change in the human lineage
Eucharist Kun, Mashaal Sohail, Vagheesh M. Narasimhan

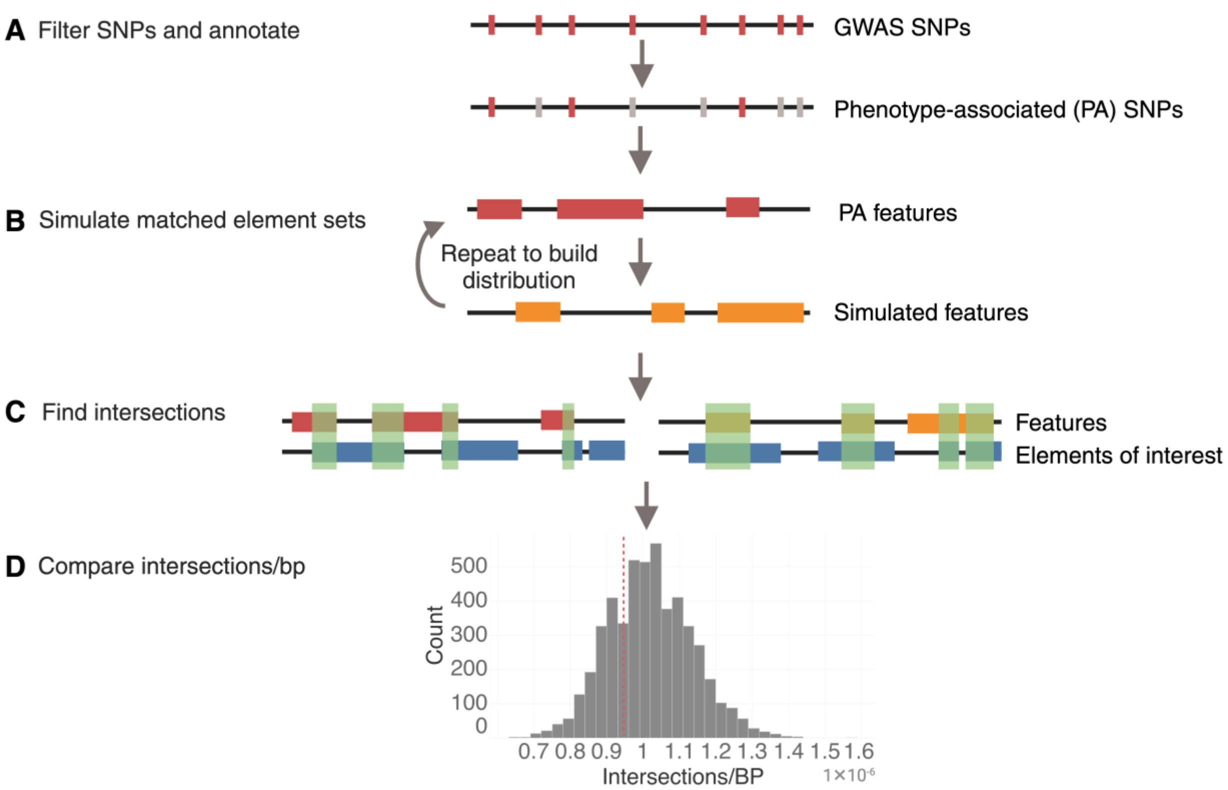

Figure S1. Genetic Feature Overlap Enrichment Pipeline (HARE) overview. **Related to STAR Methods**. **A**) Significant GWAS SNPs are mapped to genes. **B**) A simulation of length matched genes is created. **C**) Both the phenotype gene set and simulation set are intersected with the annotation of interest. **D**) More simulations are created and intersected with the annotation set until a background distribution is generated. The phenotype gene set intersection value is compared to this background distribution and a p-value is calculated. (Image by Olivia Smith, sourced from paper previously published in Journal of Open-Source Software (JOSS)[29], used under CC BY 4.0)

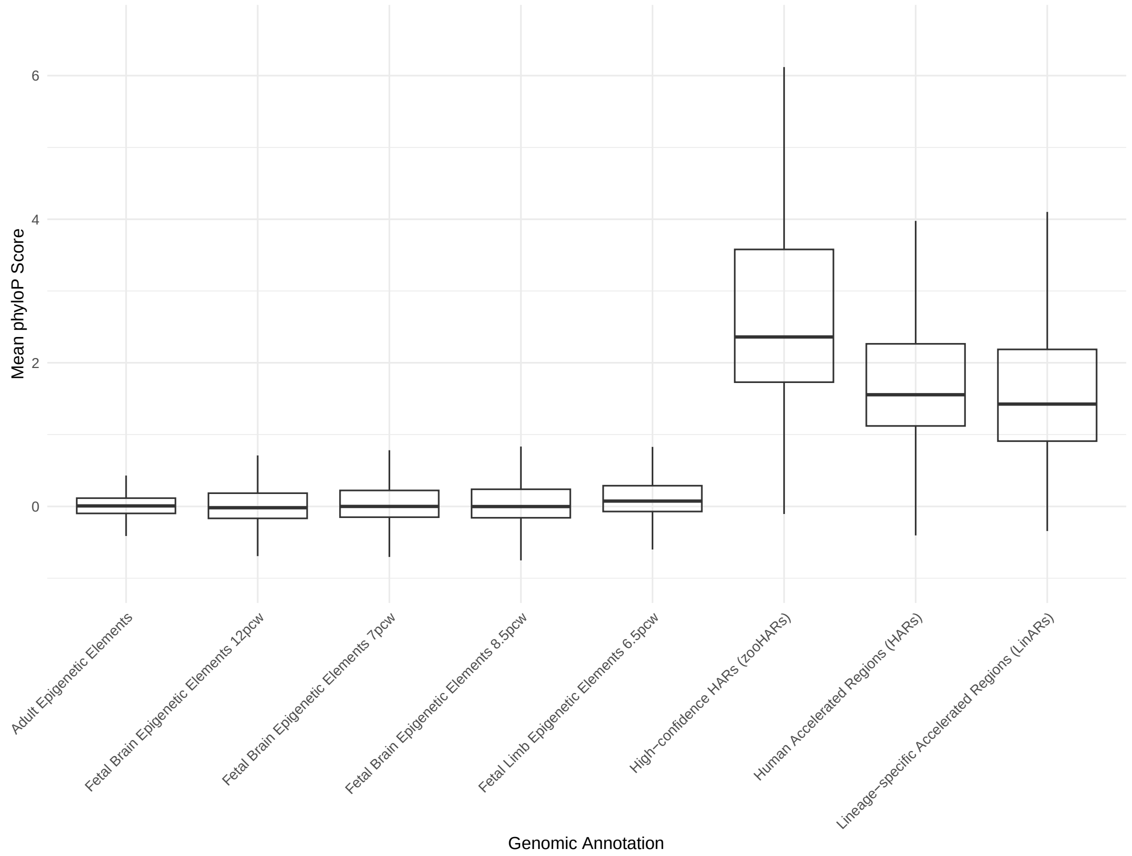

Figure S2. A boxplot of phyloP score distributions for HGEPs and HARs. Related to Figure 2. PhyloP score distributions showing the overall level of mammalian conservation for each genomic annotation. Higher scores means that a region is more conserved.

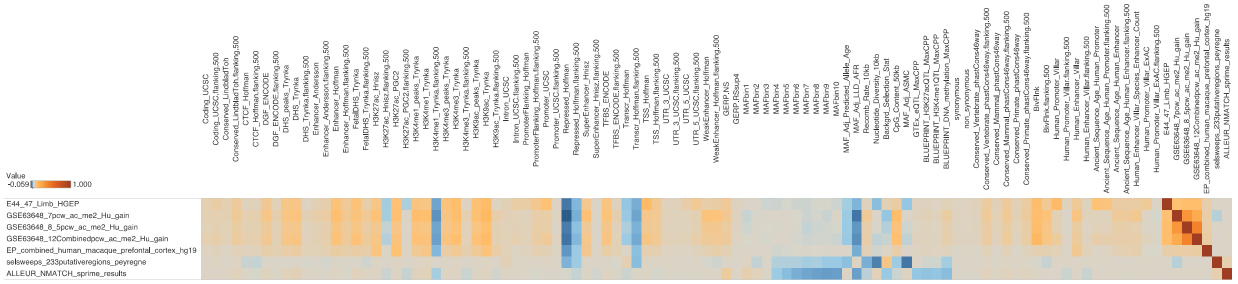

Figure S3. Correlations between baselineLDv2.2 annotations and genomic annotations used in S-LDSC analysis. Related to Figures 2 and 3 and STAR Methods.

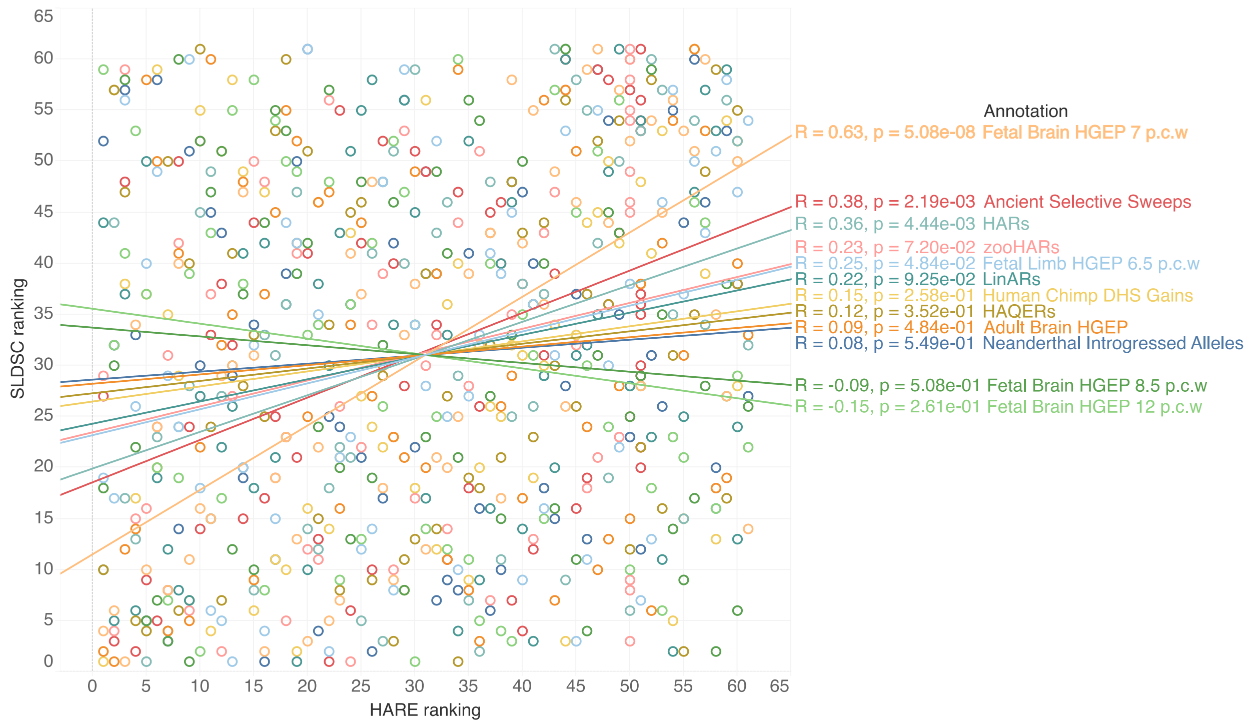

Figure S4. Scatterplot of enrichment rankings for S-LDSC and HARE across each annotation. Related to Figure 3 and Table S6. The Pearson correlation for each annotation and subsequent p-value is displayed to the right of the plot.

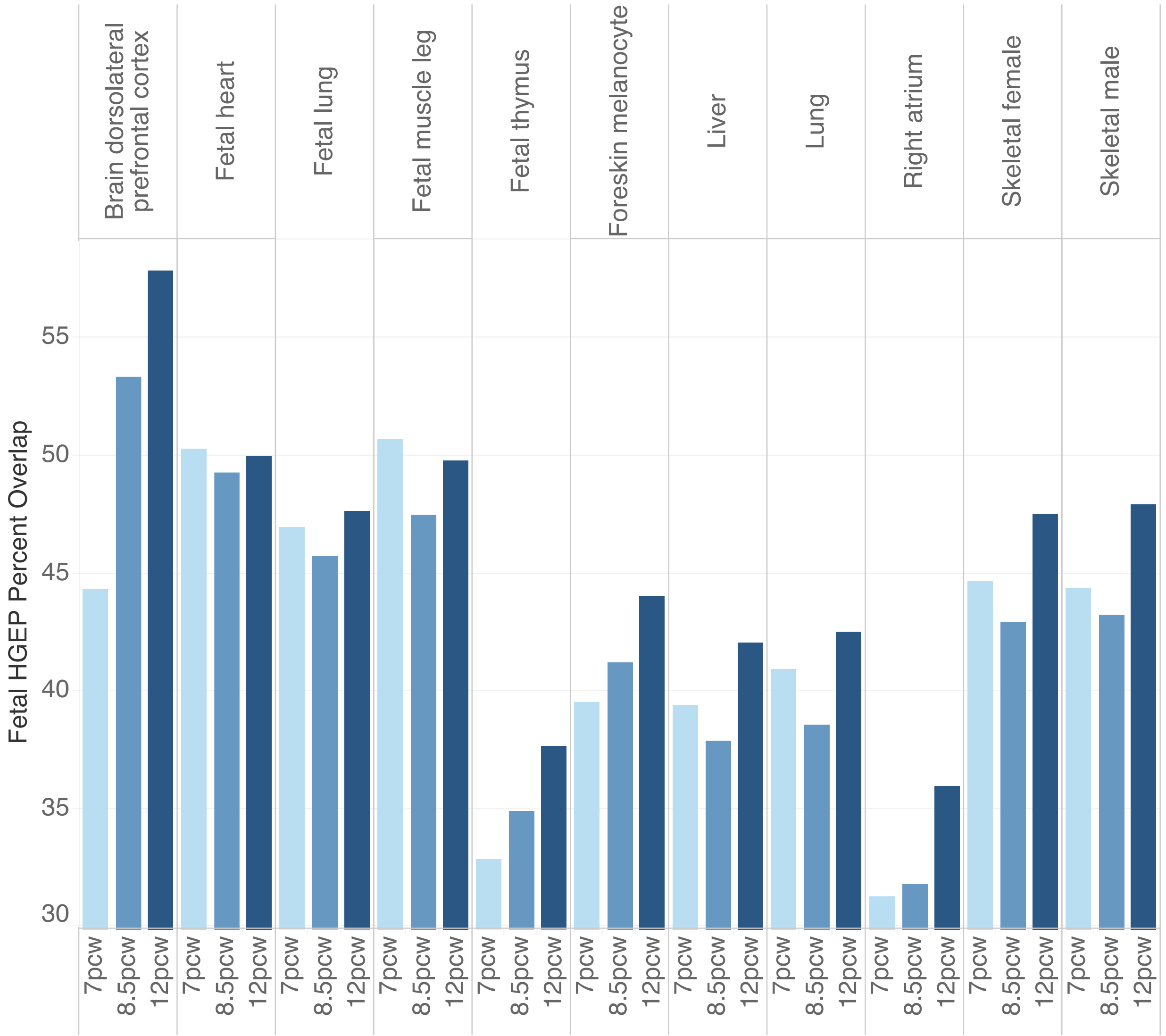

Figure S5. Comparison of base pair overlap. Related to Figure 3. Shared overlap of base pairs between enhancers and promoters of various tissues with HGEP in the brain at various fetal time points. A total of 11 tissues obtained from various origins from the Epigenome Roadmap 25 state model were analyzed. The percent overlap is calculated as the number of bases that overlap between each enhancer/promoter annotation with the Epigenome Roadmap tissue divided by the total length of the enhancer/promoter annotation - i.e. (base pair overlap of 7 p.c.w and E066 (liver) / length of 7 p.c.w).

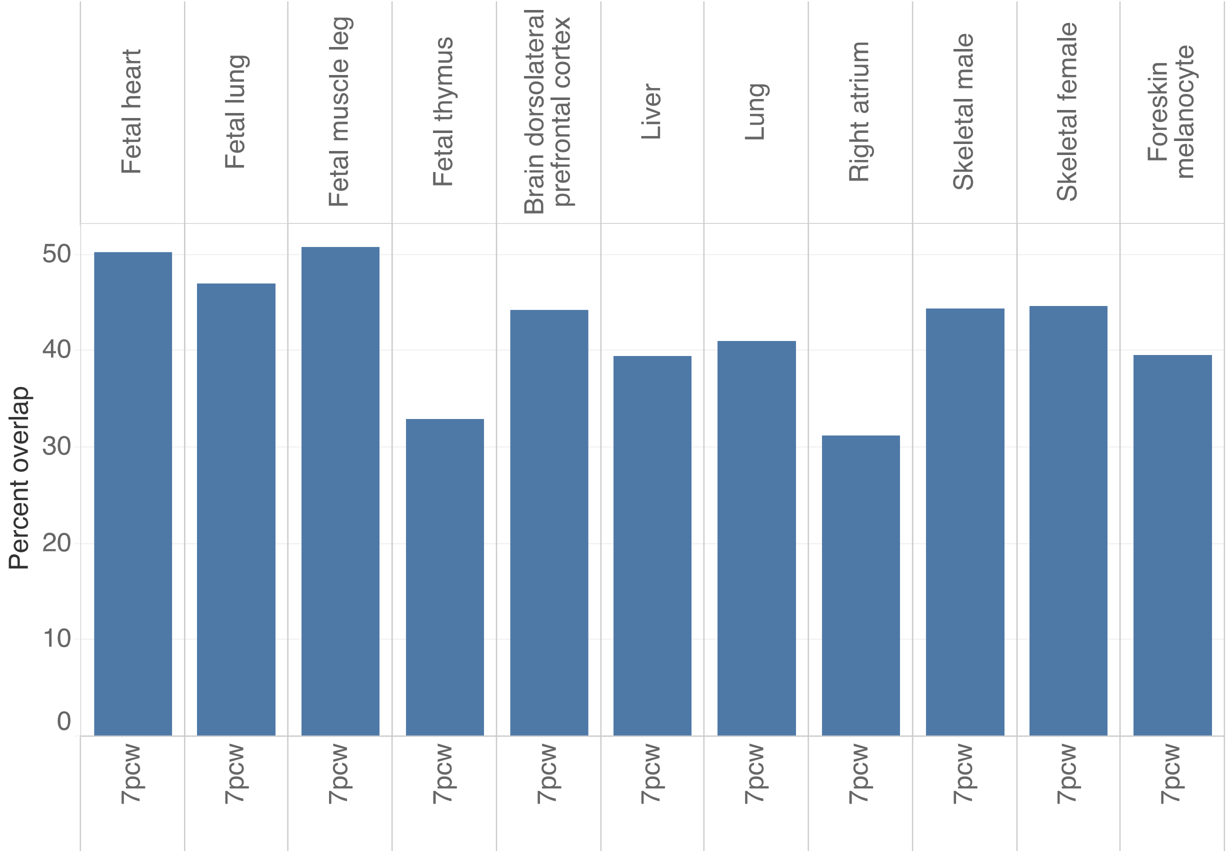

Figure S6. Shared overlap of base pairs between enhancers and promoters. Related to Figure 3. Overlap of various tissues with HGEP in the brain at 7 p.c.w only.

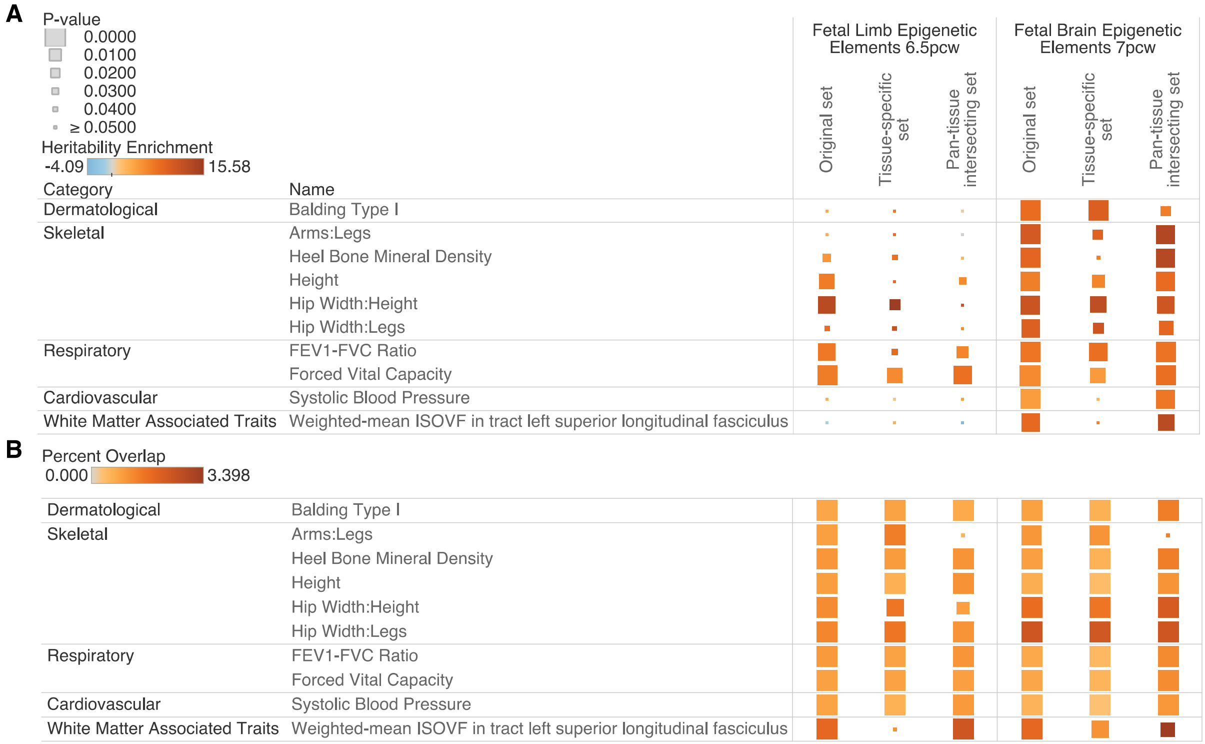

Figure S7. **Comparison of enrichment across fetal-gained enhancers and promoters. Related to Figure 3**. **A)** Heritability enrichment and **B)** gene overlap enrichment across fetal-gained enhancers and promoters.at 6.5 p.c.w in the limbs and 7 p.c.w in the brain when intersected with other tissue types. Heritability and gene overlap enrichment carried out on the original set shown on the left column followed by cross-tissue enhancers and promoters then brain-specific enhancers and promoters. All traits shown are significant in the leftmost column at FDR-adjusted p-value < 0.05. Larger squares represent lower p-values.

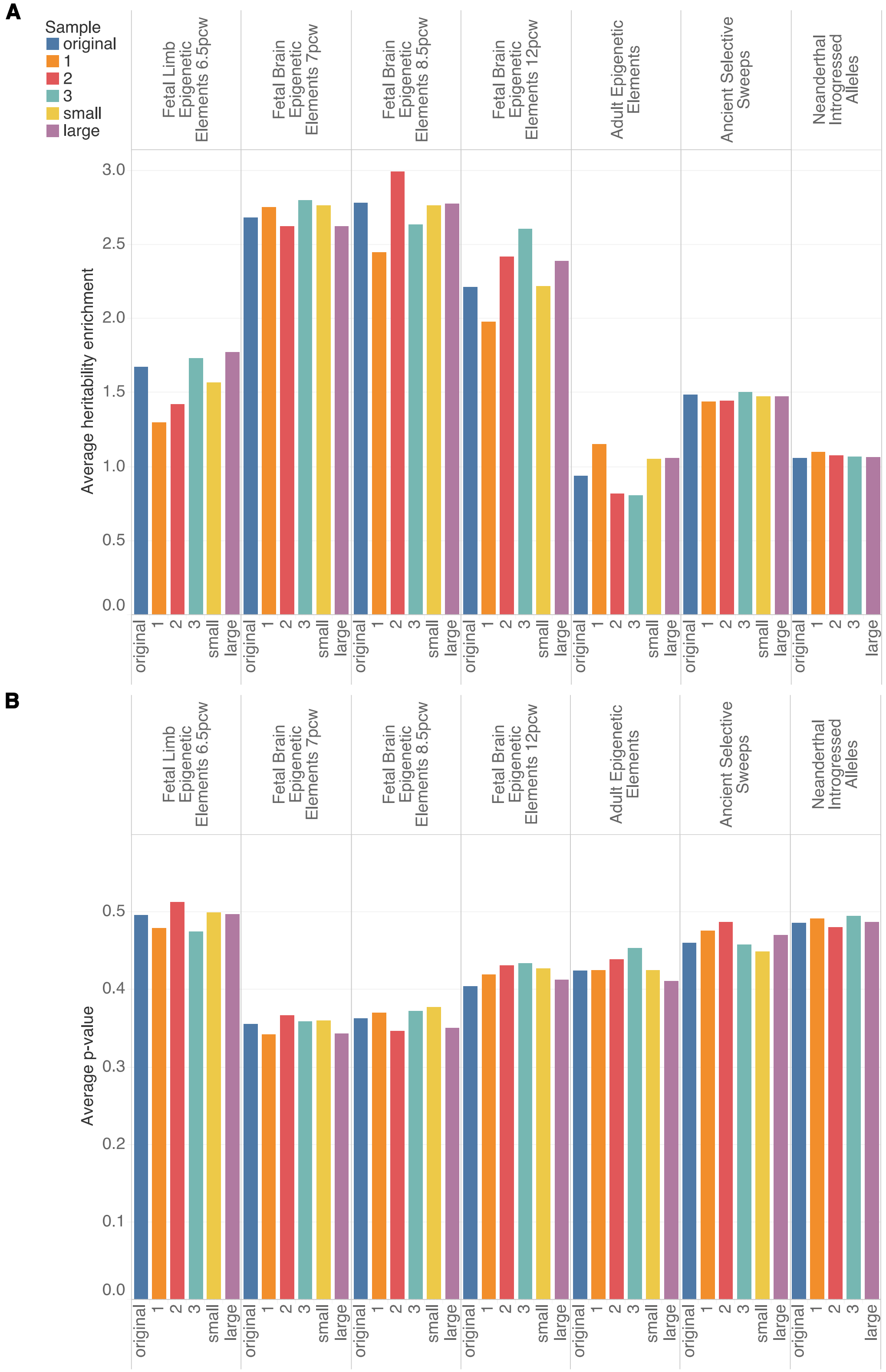

Figure S8. Comparison of S-LDSC results across modified annotations. Related to Figure 3. Average **(A)** heritability estimates and **(B)** p-values across all annotations with their modified forms. These include 3 random subsets each equal to 90% of the original annotation as well as

versions where genomic regions in the annotations were lengthened by 5% on each end or shortened by 5% on each end. For introgressed alleles, only a lengthened version was included as each entry in the annotation only spans a single nucleotide and could not be shortened.

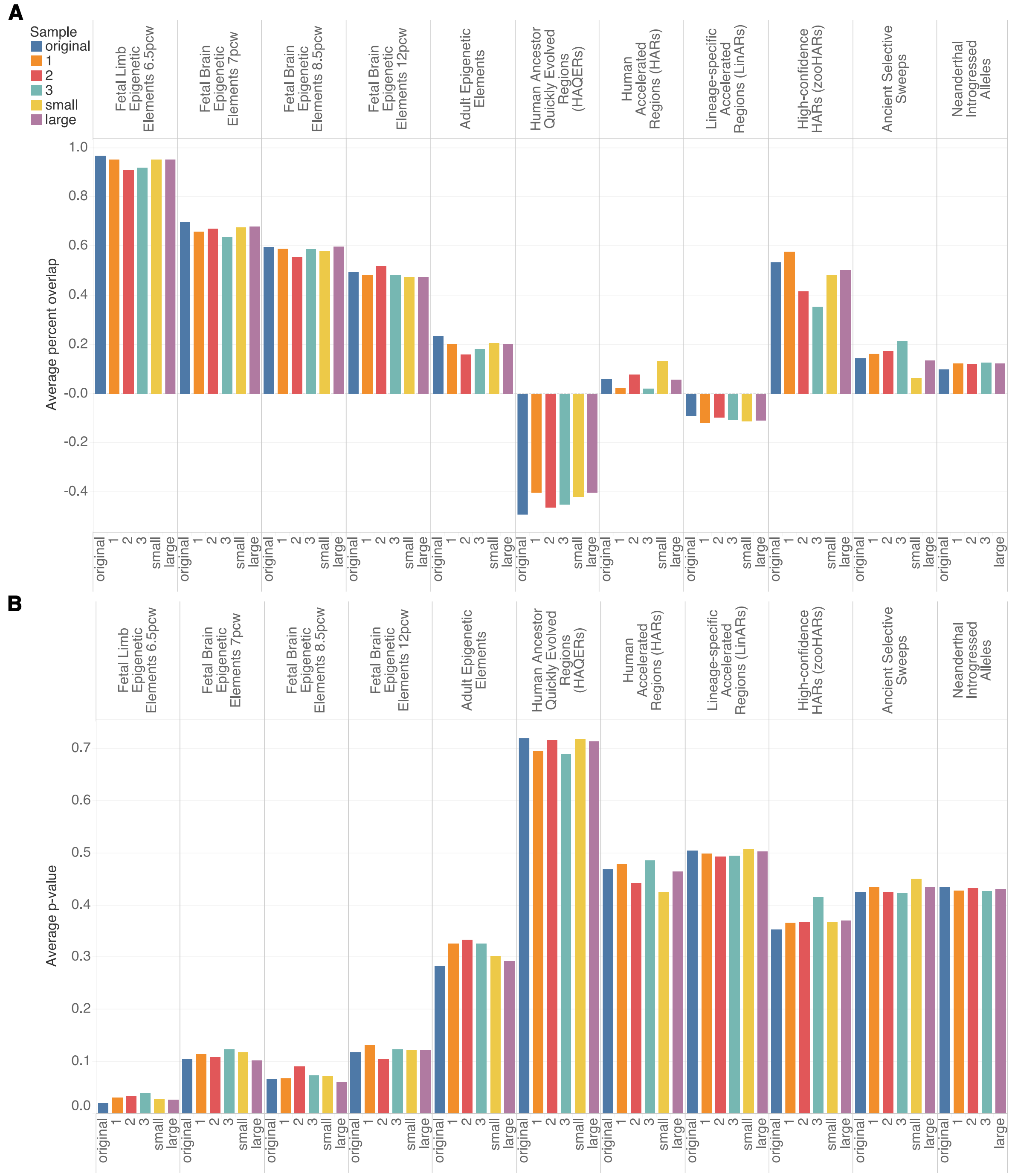

Figure S9. Comparison of HARE results across modified annotations. Related to Figure 3. Average **(A)** gene overlap estimates and **(B)** p-values across all annotations with their modified forms. These include 3 random subsets each equal to 90% of the original annotation as well as
versions where genomic regions in the annotations were lengthened by 5% on each end or shortened by 5% on each end. For introgressed alleles, only a lengthened version was included as each entry in the annotation only spans a single nucleotide and could not be shortened.

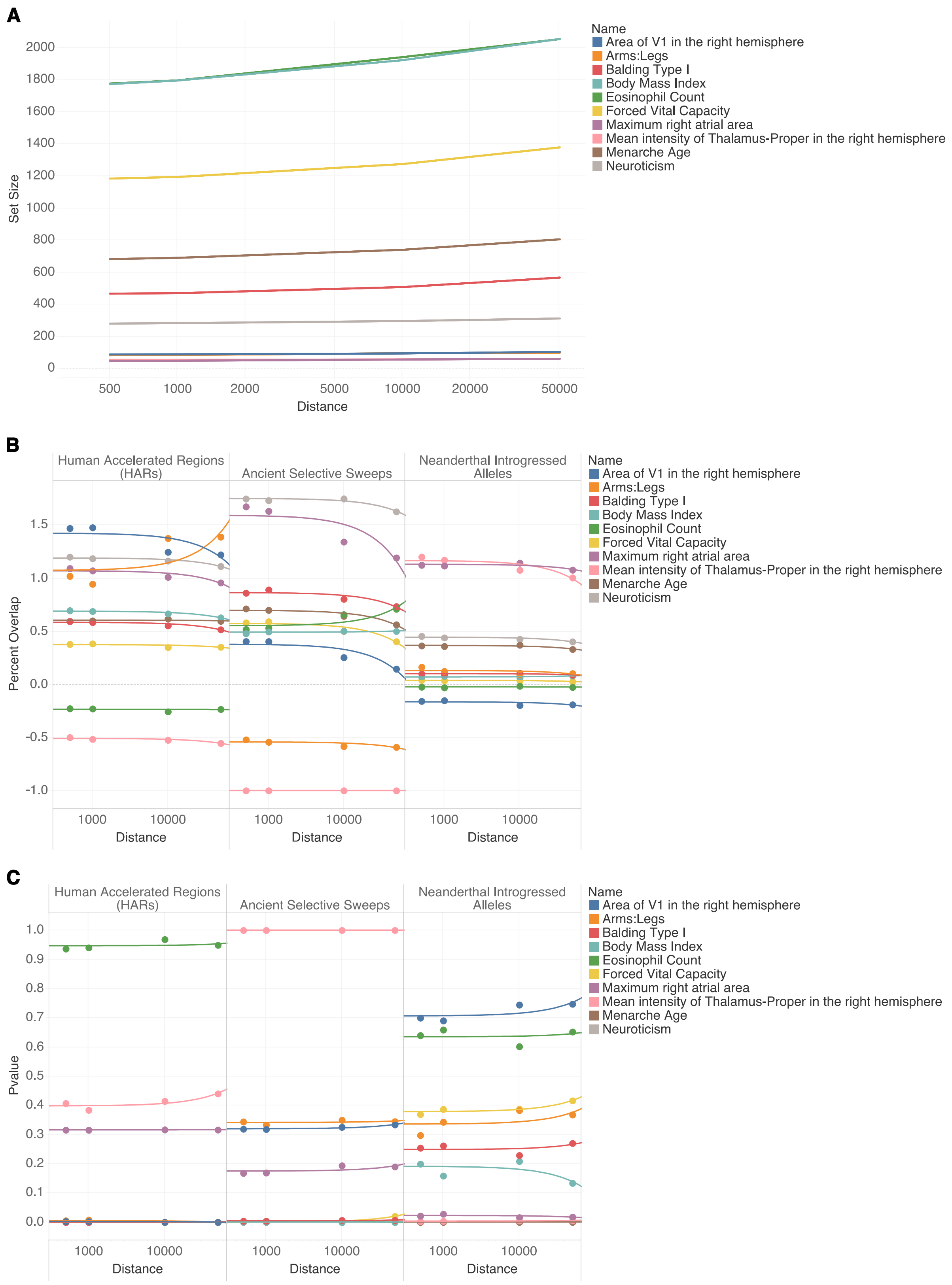

Figure S10. HARE distance threshold metrics. Related to Figure 3. A) Comparison of phenotype gene set size for 10 traits as distance threshold increases. **B)** Percent overlap for 10

traits across 3 annotations as a product of distance threshold. **C**) P-value for 10 traits across 3 annotations as a product of distance threshold.

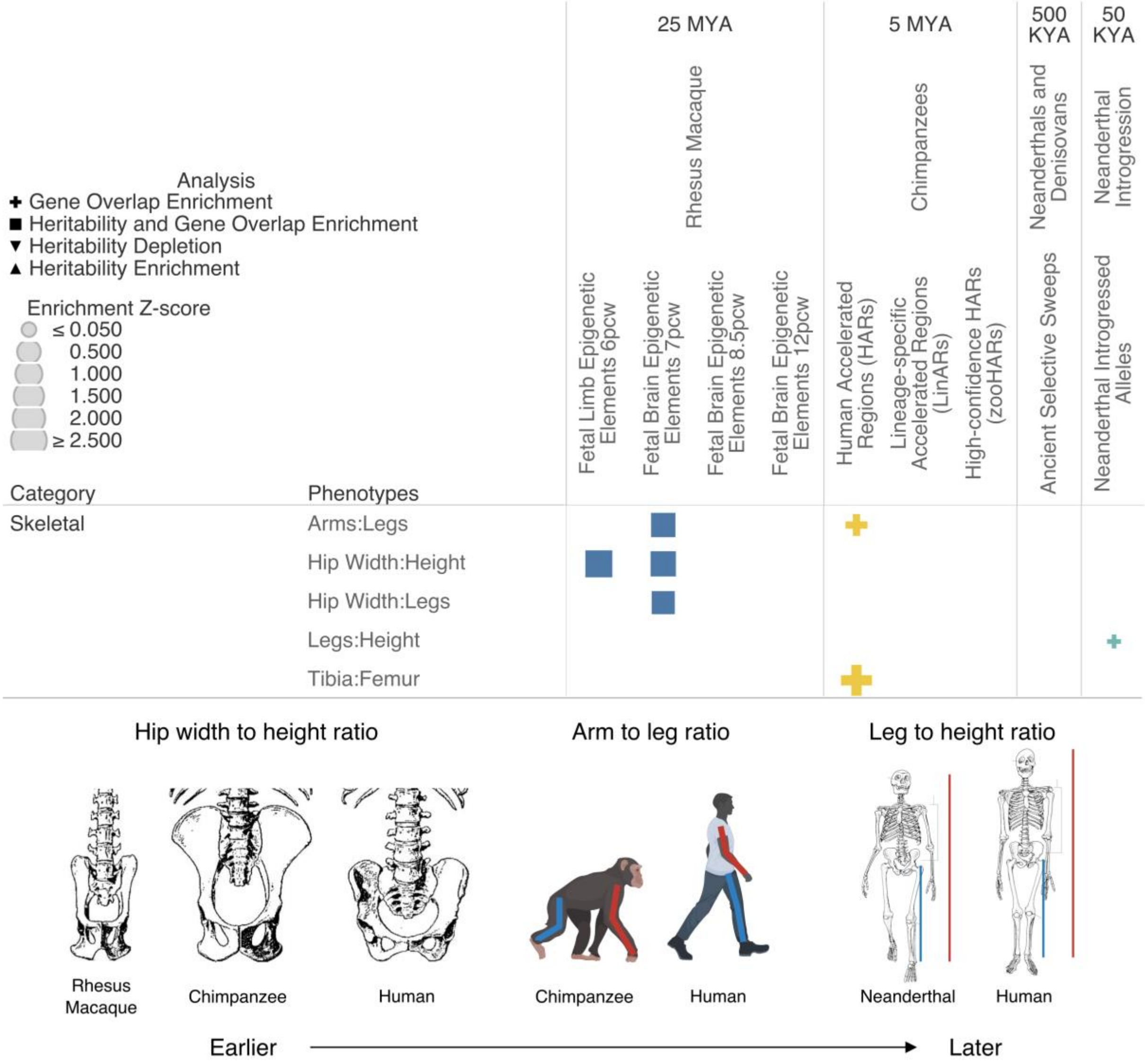

Figure S11. Skeletal trait differences and enrichments across species. Related to Figure 3. Skeletal traits that are significantly enriched in this analysis are shown in the top half of this figure while the corresponding fossil record and skeletal morphology comparisons between humans and other primate species are shown in the bottom half
